# Supplementary material for: Impact of gallbladder hypoplasia on hilar hepatic ducts in biliary atresia
Source: Commun Med (Lond). 2024 Jun 11;4:111. doi: 10.1038/s43856-024-00544-5 (PMC11166647; doi:10.1038/s43856-024-00544-5)
Supplement: Supplementary file 2 — Supplementary Information [file 43856_2024_544_MOESM2_ESM.pdf]

Supplementary Figures and Figure Legends

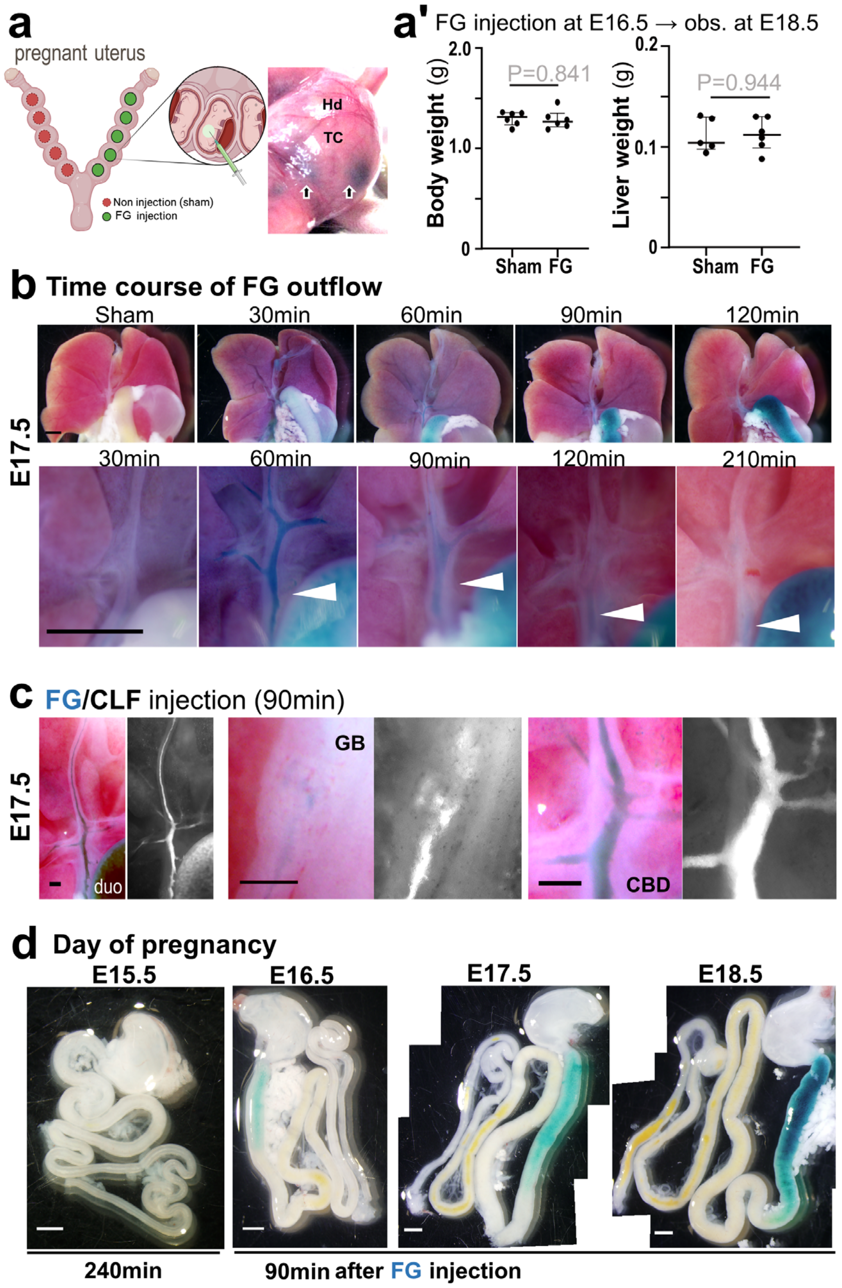

Supplementary Fig. 1. A method to visualize the fetal bile flow with the naked eye by using Fast green FCF (FG), a non-toxic contrast agent

a-a', Fast green FCF (FG) administered intraperitoneally to the intrauterine embryos, which can be defined through the uterus walls (black arrows). In **a'**, embryos, injected with FG at embryonic days (E)16.5, showed no significant changes in both the body weight (n = 6 for each FG or sham group, maternal: n=1) and liver weight (sham: n = 5, FG: n=6, maternal: n=1) at E18.5. Data were presented as median with interquartile range. Statistical comparisons were performed using Two-tailed unpaired Welch's t-test. **b**, Time course of bile flow in intrauterine embryos showing transient FG signals in the hepatic lobule at 30-120 min and in the common bile duct at 30-210 min after injection (arrowhead). **c**, FG and cholyl-lysyl fluorescein (CLF; fluorescein-labeled bile acid) signals completely overlap in the extrahepatic duct. The left panel corresponds to the lower magnification of the two right panels. **d**, Intrauterine embryos at E15.5 to E18.5 injected with FG, showing stage-dependent FG excretion into the duodenum (note the onset of the bile flow at E15.5-E16.5). Hd, head; TC, thoracic cavity; duo, duodenum; GB, gallbladder; CBD, common bile duct. Scale bars, 1mm (**b, d**), 200 µm (**c**).

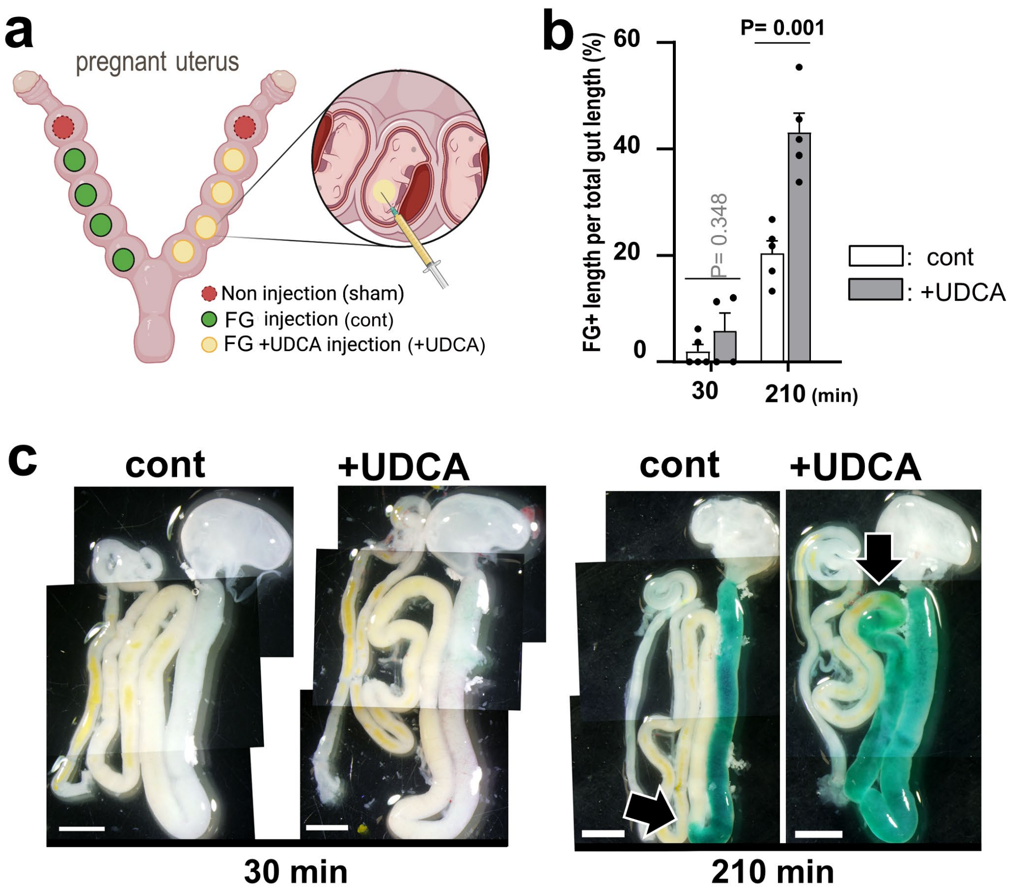

Supplementary Fig. 2. Estimating fetal bile flow as the reachable distance of Fast green FCF (FG)+ signals along the fetal gut

a, Experimental design to estimate the level of embryonic (E) 17.5 fetal bile flow by using Fast green FCF (FG) in combination with ursodeoxycholic acid (UDCA; an agent that increases bile acid flow and promotes the secretion of bile acids). **b, c**, The reachable distance of FG+ signals (arrows) along the fetal intestine after injection with or without UDCA (100µg /g embryonic BW), together with FG. In **b**, UDCA significantly increases FG+ length per total gut length (30min: control: n=5, +UDCA: n=4 maternal: n=1, 210min: control: n=5, +UDCA: n=5, maternal: n=1, mean ± s.e.m; two-tailed unpaired Welch's t-test). Scale bars, 2 mm (**c**).

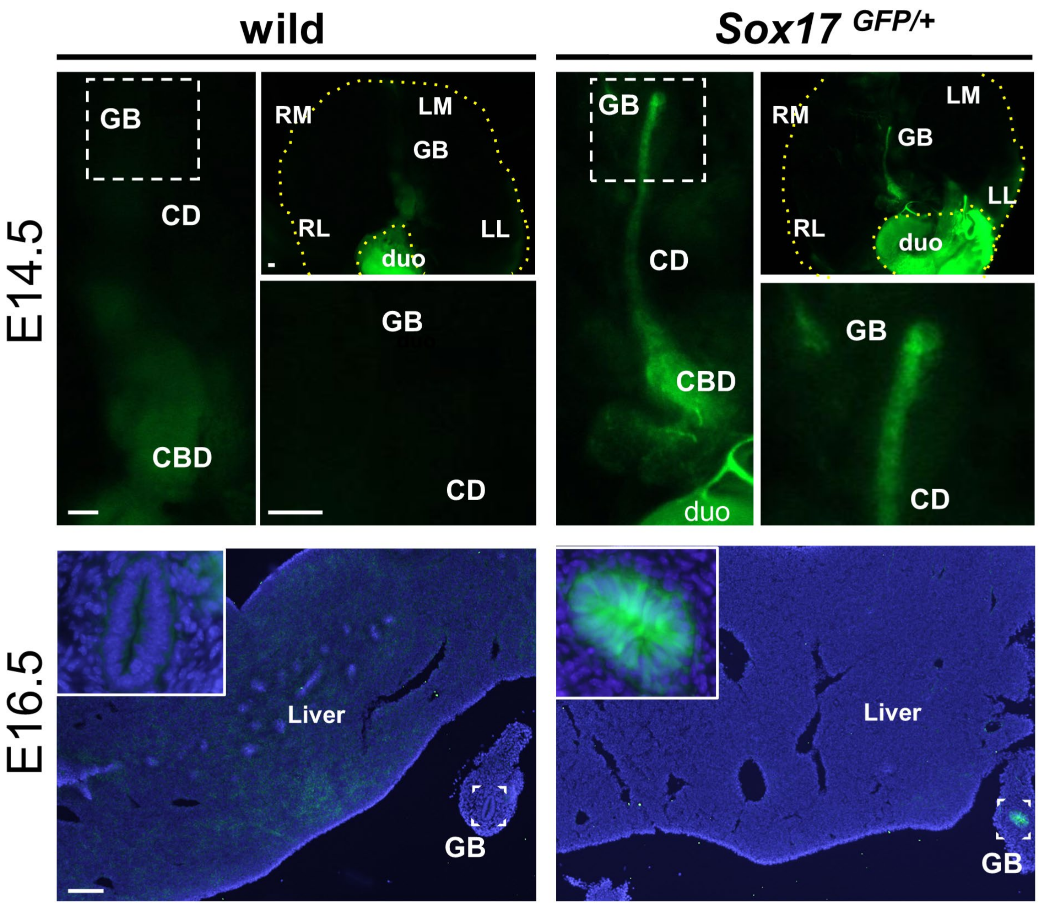

Supplementary Fig. 3. Restricted expression of SOX17 in the gallbladder walls in the developing hepatobiliary tissues

a, b, *Sox17*- Green fluorescent Protein (GFP; green) signals in the whole liver of *Sox17* $^{GFP/+}$ embryos at embryonic days (E) 14.5 (whole mount view) and E16.5 (section), showing high expression in the gallbladder-cystic duct wall, but not in the liver lobule (dotted outline). Note some GFP signals from the arterial endothelial cells around the common bile duct. CD, cystic duct; GB, gallbladder; CBD, common bile duct; LM, left medial lobe; LL, left lateral lobe. RM, right medial lobe; RL, right lateral lobe. duo. duodenum, Scale bars, 100 µm.

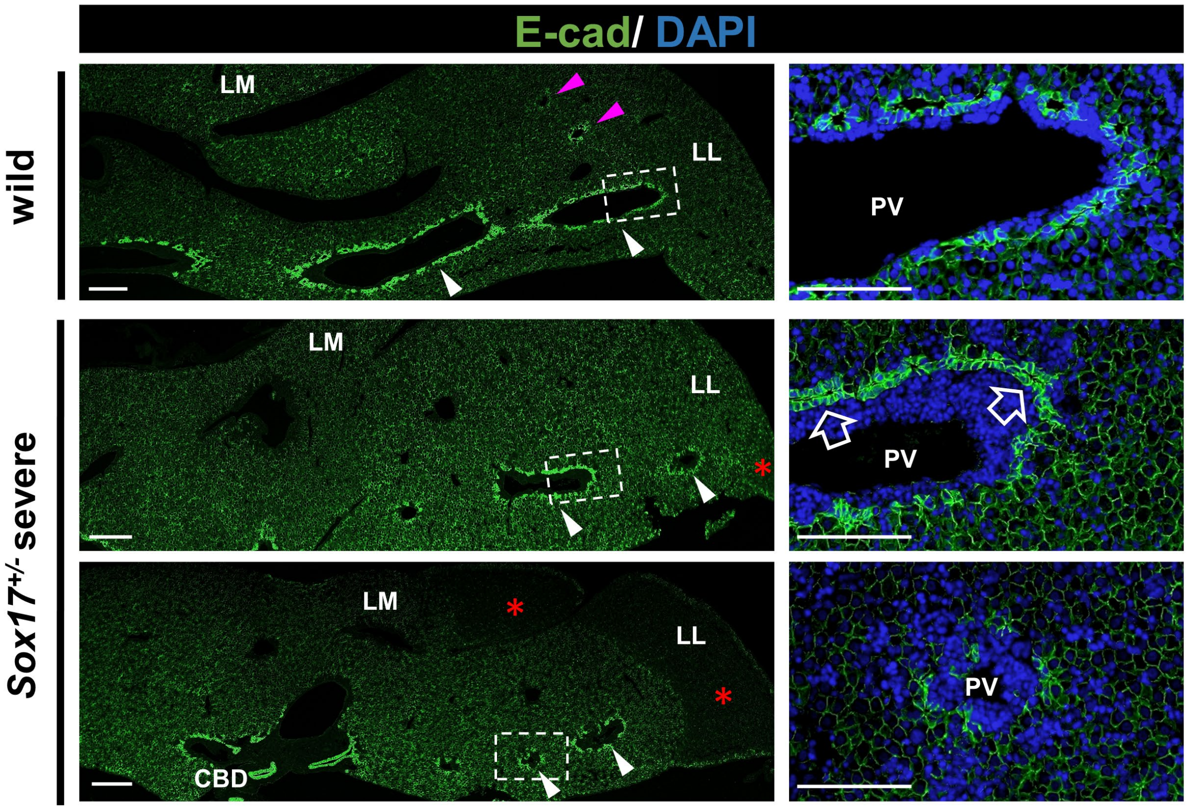

Supplementary Fig. 4. Defective formation of intrahepatic bile ducts (IHBD) in *Sox17*$^{+/-}$ embryo
Anti-E-cadherin (E-cad) immunostaining of the sections in the left lateral liver lobe of *Sox17*$^{+/-}$ and wild-type littermates at embryonic days (E)18.5. In wild-type embryos, some proximal intrahepatic bile ducts (IHBDs) (white arrowheads) form a well-defined open lumen and branch into small bile trees towards the peripheral margin (magenta arrowheads). In *Sox17*$^{+/-}$ embryos, proximal IHBDs showed poor development of luminal structure (open arrows) with no peripheral extension of the bile trees. Red asterisk, hepatic degeneration; CBD, common bile duct; PV, portal vein; LM, left medial lobe; LL, left lateral lobe. Scale bars, 200μm (left) and 100μm (Right).

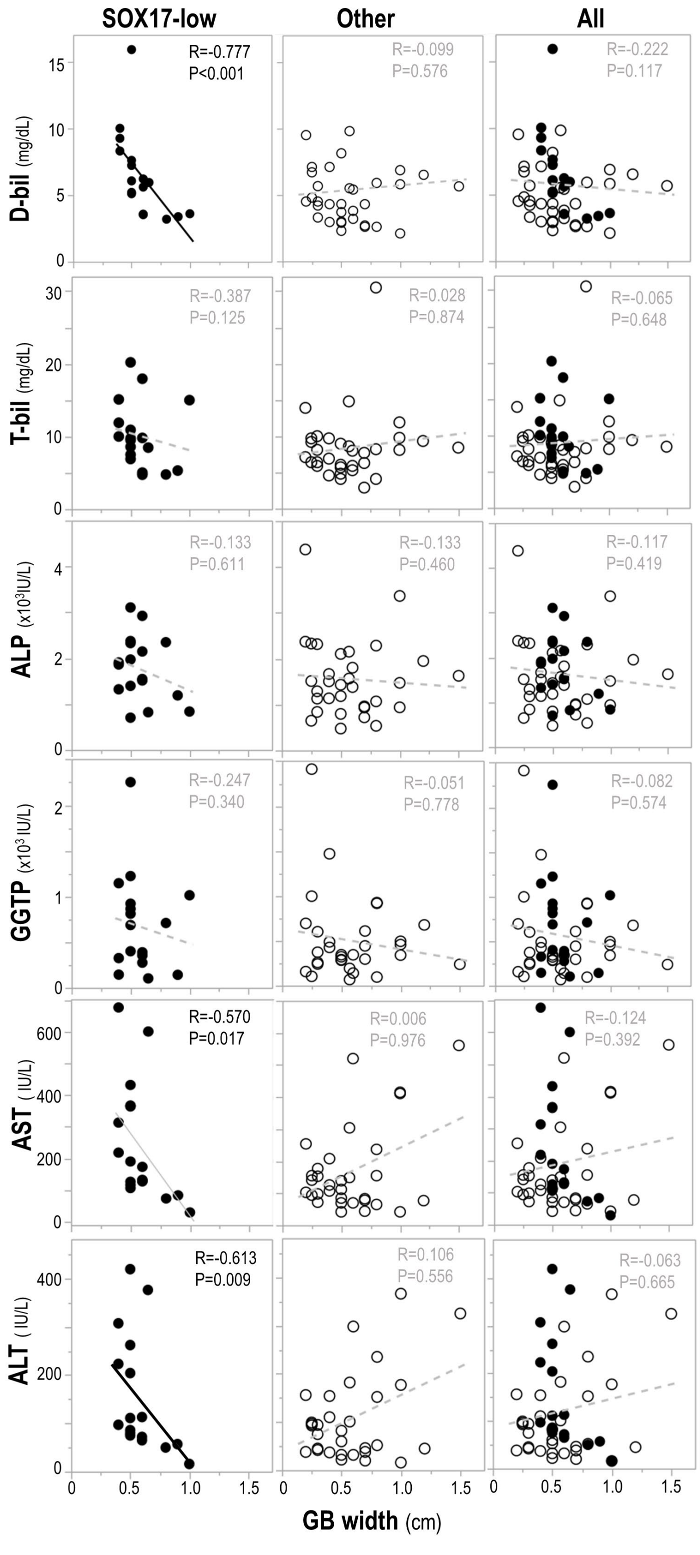

Supplementary Fig. 5a. Correlations of serum markers with gallbladder (GB) width in human biliary atresia (BA). Spearman correlation coefficients between gallbladder width (x-axis: gallbladder width [cm]) and serum markers (y-axis: direct bilirubin [D-bil, mg/dl], total bilirubin [T-bil, mg/dL], alkaline phosphatase [ALP, × 10^3 IU/l], γ-glutamyl transpeptidase [GGTP, × 10^3 IU/l], aspartate aminotransferase [AST, IU/l], and alanine aminotransferase [ALT, IU/l]) in SOX17-low (left), other (middle), and all BA groups. Solid and open dots, SOX17-low and other groups, respectively (black solid line, $p < 0.01$; gray solid line, $0.01 \leq p < 0.05$; gray broken line, $p \geq 0.05$) (SOX17-low: n=17, Other: n=34, All: n=51).

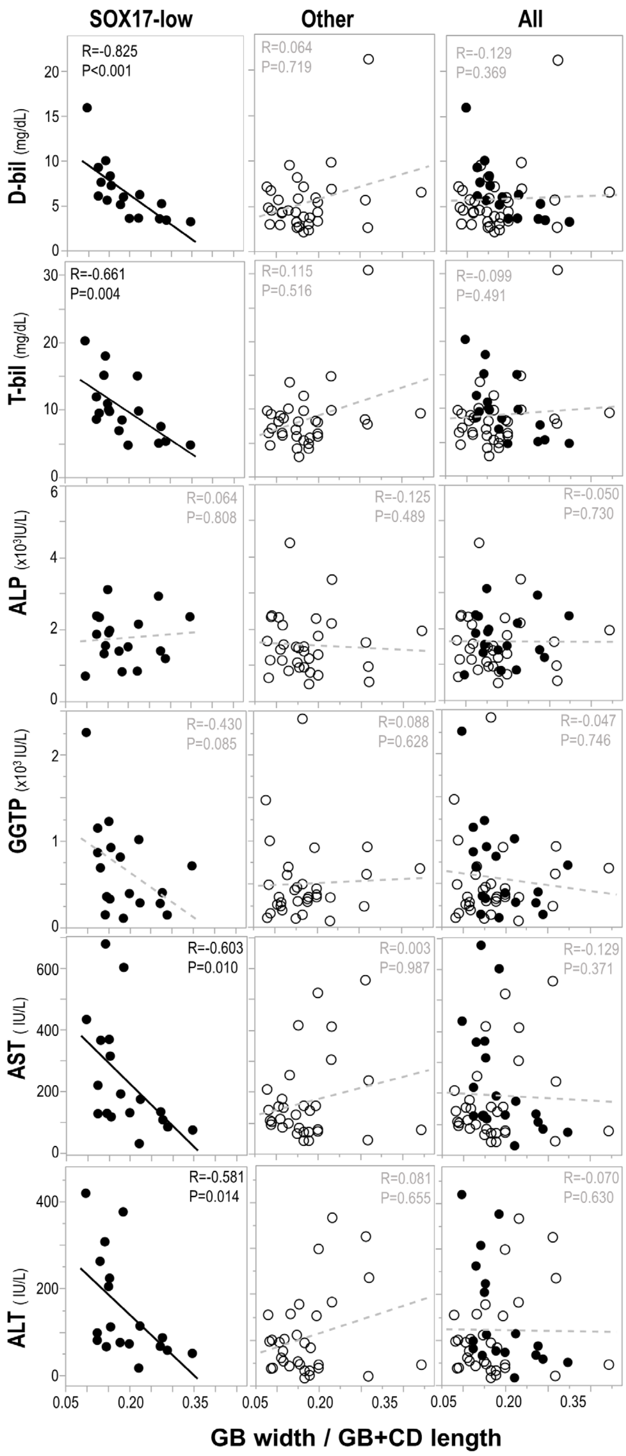

Supplementary Fig. 5b. Correlations of serum markers with relative gallbladder (GB) width per gallbladder-cystic duct (GB+CD) length in human biliary atresia (BA). Spearman correlation coefficients between relative gallbladder width per gallbladder-cystic duct length (x-axis: GB width / GB+CD length) and serum markers (y-axis: direct bilirubin [D-bil, mg/dl], total bilirubin [T-bil, mg/dL], alkaline phosphatase [ALP, × 10^3 IU/l], γ-glutamyl transpeptidase [GGTP, × 10^3 IU/l], aspartate aminotransferase [AST, IU/l], and alanine aminotransferase [ALT, IU/l]) in SOX17-low (left), other (middle), and all BA groups. Solid and open dots, SOX17-low and other groups, respectively (black solid line, $p < 0.01$; gray solid line, $0.01 \leq p < 0.05$; gray broken line, $p \geq 0.05$). (SOX17-low: n=17, Other: n=34, All: n=51)

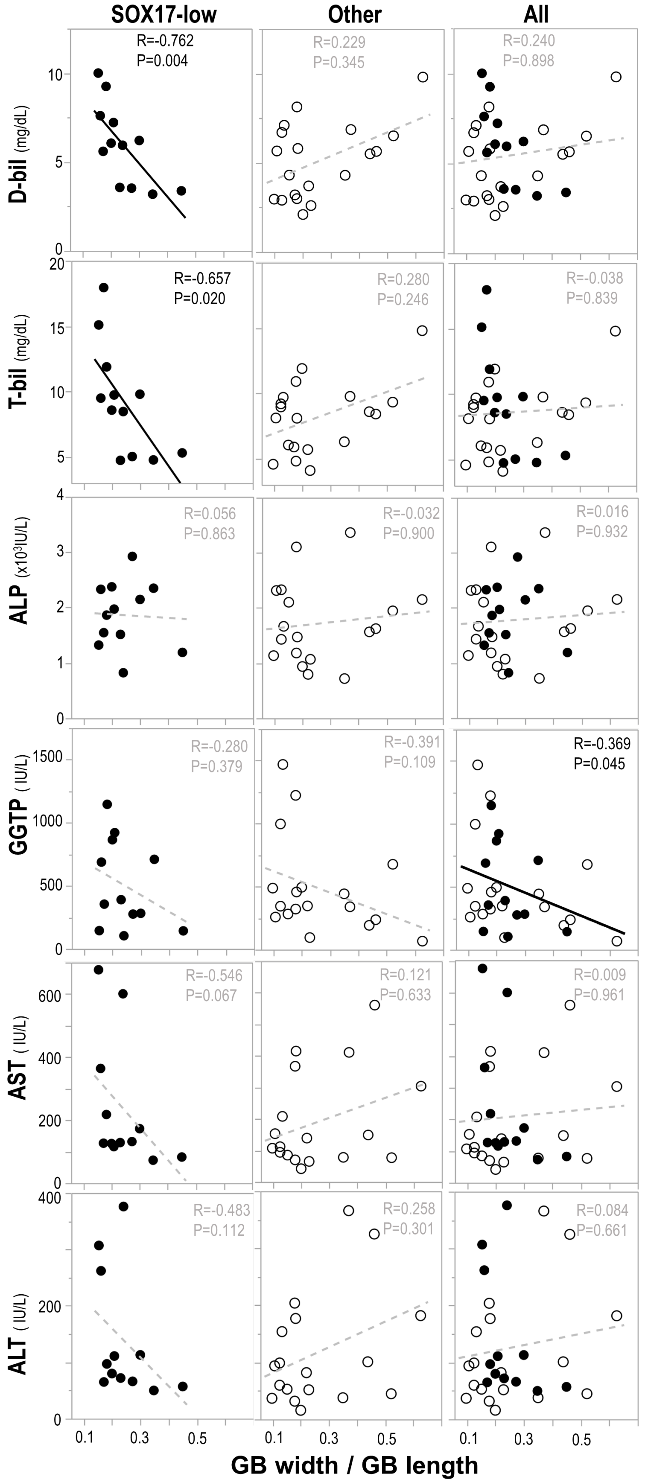

Supplementary Fig. 5c. Correlations of serum markers with relative gallbladder (GB) width per GB length in human biliary atresia (BA). Spearman correlation coefficients between relative gallbladder width per gallbladder length (x-axis: GB width/GB length) and serum markers (y-axis: direct bilirubin [D-bil, mg/dl], total bilirubin [T-bil, mg/dL], alkaline phosphatase [ALP, $\times 10^3$ IU/l], γ-glutamyl transpeptidase [GGTP, $\times 10^3$ IU/l], aspartate aminotransferase [AST, IU/l], and alanine aminotransferase [ALT, IU/l]) in SOX17-low (left), other (middle), and all BA groups. Solid and open dots, SOX17-low and other groups, respectively (black solid line, $p < 0.01$; gray solid line, $0.01 \le p < 0.05$; gray broken line, $p \ge 0.05$). (SOX17-low: n=12, Other: n=18, All: n=30)

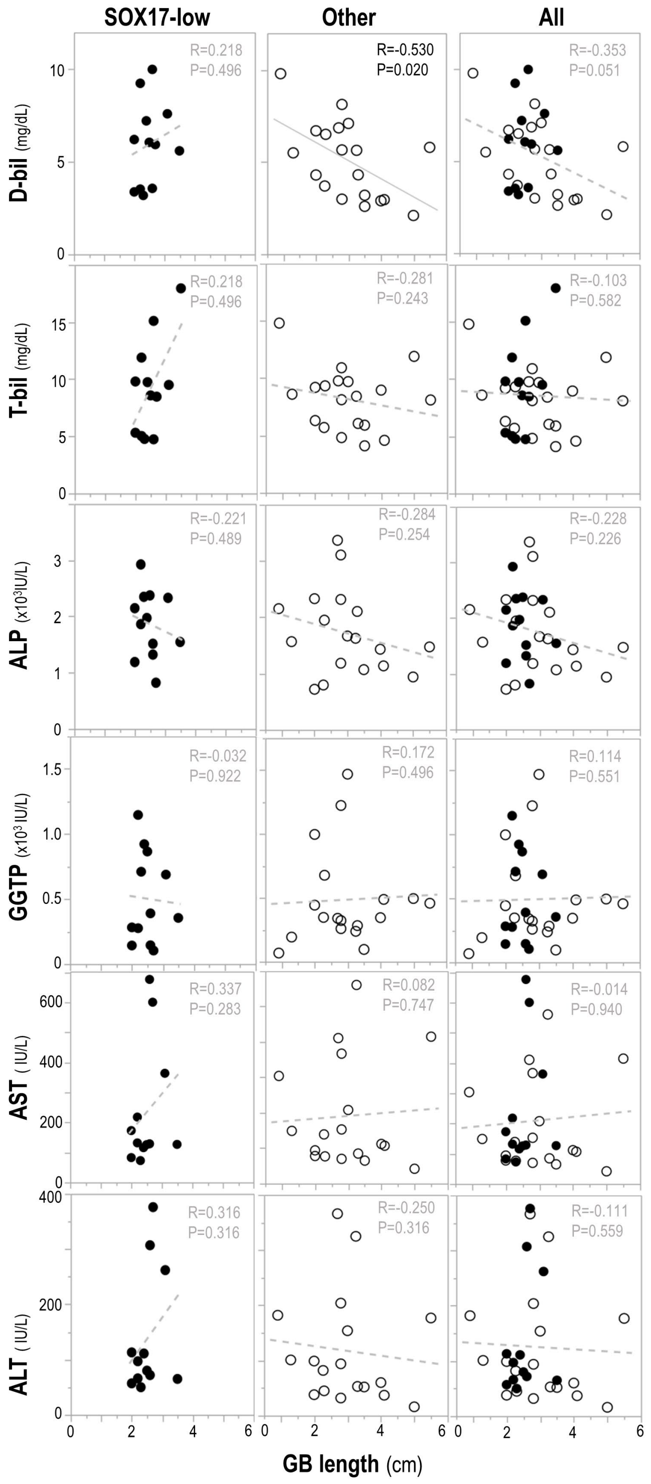

Supplementary Fig. 5d. Correlations of serum markers with gallbladder (GB) length in human biliary atresia (BA). Spearman correlation coefficients between gallbladder length (x-axis: gallbladder length [cm]) and serum markers (y-axis: direct bilirubin [D-bil, mg/dl], total bilirubin [T-bil, mg/dL], alkaline phosphatase [ALP, $\times 10^3$ IU/l], γ-glutamyl transpeptidase [GGTP, $\times 10^3$ IU/l], aspartate aminotransferase [AST, IU/l], and alanine aminotransferase [ALT, IU/l]) in SOX17-low (left), other (middle), and all BA groups. Solid and open dots, SOX17-low and other groups, respectively (black solid line, $p < 0.01$; gray solid line, $0.01 \leq p < 0.05$; gray broken line, $p \geq 0.05$). (SOX17-low: n=12, Other: n=18, All: n=30)

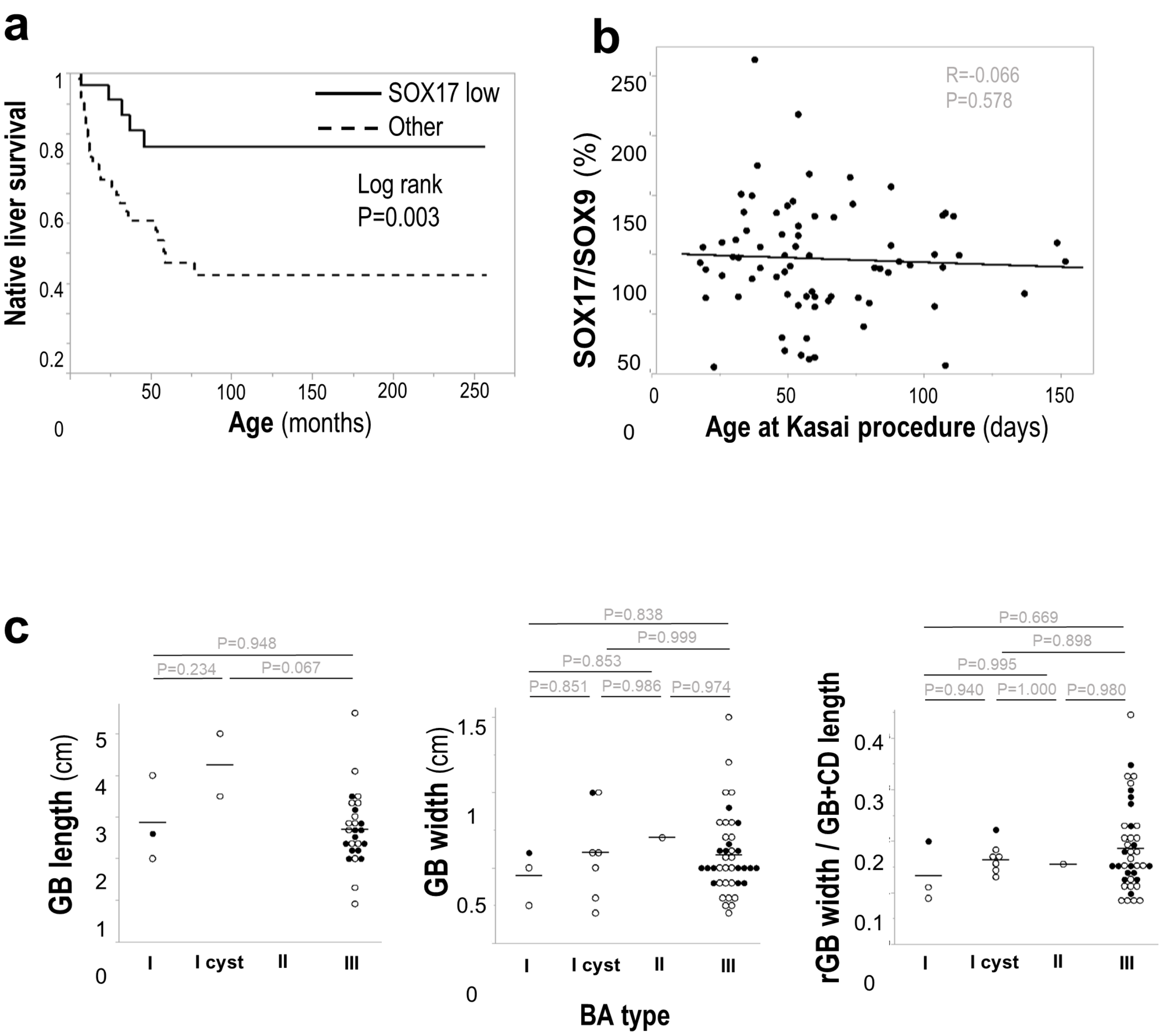

Supplementary Fig. 6. Better prognosis of SOX17-low cases in native liver survival after Kasai surgery
a, Native liver survival of SOX17-low biliary atresia. Kaplan–Meier curves of native liver survival (x-axis; months after Kasai surgery) in SOX17-lower (solid line) and -other (broken line) groups, compared by the log-rank test (SOX17-low: n=24, Other: n=49). **b**, Correlation of SOX17/SOX9 index with age at Kasai procedure. Spearman correlation coefficients between SOX17/SOX9 (x-axis: SOX17/SOX9) and age at Kasai procedure (y-axis: age at Kasai procedure [days] (n=74). **c,** Morphological analysis of BA types. Dot plots of the length (I, I cyst, II, III: n=3, 2, 0, 25, respectively), width of the presumptive gallbladder region (GB) (I, I cyst, II, III: n=3, 7, 1, 40, respectively) and relative gallbladder width per gallbladder-cystic duct length [rGBW/GB+CD length] (y-axis) (I, I cyst, II, III: n=3, 7, 1, 40, respectively) in BA types. Data were analyzed by Tukey's honestly significant difference test. Solid and open dots indicate SOX17-low and other group respectively.

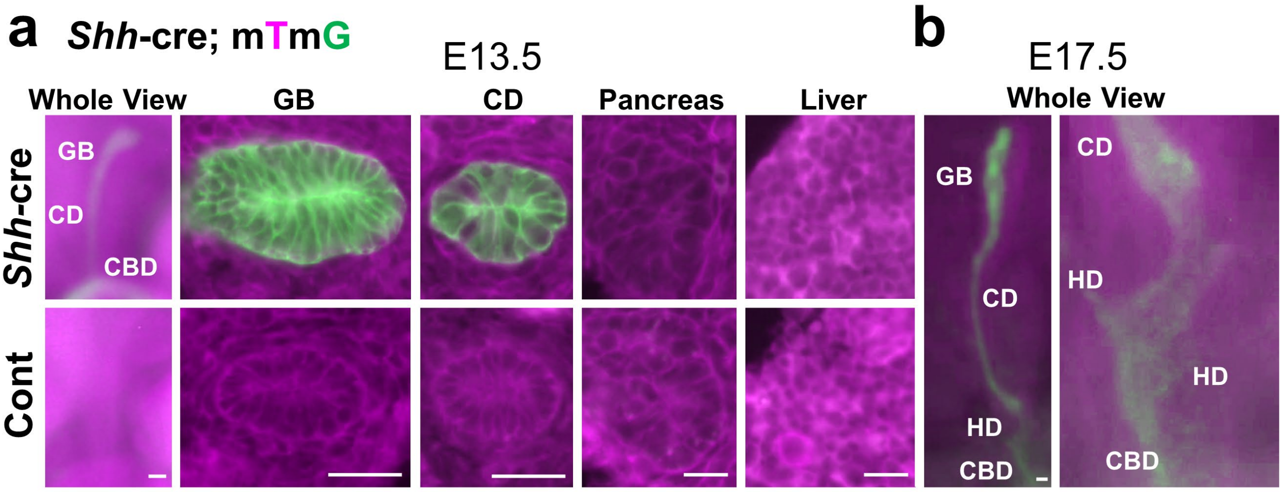

Supplementary Fig. 7. Extrahepatic duct-specific deletion of *Sox17* flox allele by *Shh*-cre mice

a, b, High expression of Green fluorescent Protein (GFP; green) in gallbladder and cystic duct region of *Shh*-cre; *ROSA*^{mTmG} embryos at embryonic day (E) 13.5 (**a**) and E17.5 (**b**). No GFP expression is detectable in the pancreas and liver, in addition to no GFP expression in the control *ROSA*^{mTmG} embryos. CBD, common bile duct; CD, cystic duct; GB, gallbladder; HD, hepatic duct. Scale bar: 100 µm.

Supplementary table. 1. Clinical and laboratory parameters of the SOX17-low biliary atresia and Others[1)]

Variable	SOX17-low (n=24)	Other (n=50)	P-value
**SOX17/SOX9 index, %**	46.4±23.1	118.9±35.6	**<0.001**
**SOX17+ cell number, %**	40.7±24.9	66.9±23.5	**<0.001**
SOX9+ cell number, %	58.9±21.1	57.6±22.7	0.846
PBG density, number/100μm	2.78±3.75	2.82±4.02	0.980
GB-CD length (cm)	3.34±0.97	3.45±1.29	0.740
GB length (cm)	2.53±0.43	3.01±1.18	0.171
GB width (cm)	0.58±0.17	0.58±0.31	0.990
rGB width/ GB+CD length	0.19±0.07	0.17±0.08	0.442
rGB width/ GB length	0.24±0.09	0.26±0.16	0.653
Gender; male:female	10:14	24:26	0.623*
Age at Kasai procedure, days	61.3±26.3	59.8±32.3	0.842
D-bil, mg/dl	6.11±2.94	5.68±3.40	0.602
D-bil, 2weeks after Kasai, mg/dL	2.06±1.44	3.67±2.41	0.060
Recovery rate of D-bil after KASAI	0.43±0.30	0.73±0.47	0.063
Cholangitis before 12m, %	55.6 (5/9)	58.8 (20/34)	1.000*
Liver transplantation before 24 months, %	15.8 (3/19)	38.6 (17/44)	0.086*
Age at liver transplantation, month	25.8±15.6	18.9±14.9	0.320
**Liver transplantation case, %**	26.1 (6/23)	54.2 (26/48)	**0.041***
BA type case[2)], number (I, I cyst, II, III)	(1,4,0,19)	(3,7,1,39)	0.945*

[1)]Quantitative data are means ± standard deviations; they were analyzed by Student's two-tailed t-tests. Nominal scales were subjected to Fisher's exact test (asterisk). Bold font indicates statistical significance ($p < 0.05$).

[2)] Biliary atresia [BA] type is used Ohi classification). D-bil, direct bilirubin; GB, gallbladder; GB-CD, gallbladder-cystic duct; PBG, peribiliary gland.
