# Supplementary material for: Impact of gallbladder hypoplasia on hilar hepatic ducts in biliary atresia
Source: Commun Med (Lond). 2024 Jun 11;4:111. doi: 10.1038/s43856-024-00544-5 (PMC11166647; doi:10.1038/s43856-024-00544-5)
Supplement: Supplementary file 3 — Description of Additional Supplementary Files [file 43856_2024_544_MOESM3_ESM.pdf]

Description of Additional Supplementary Files

File name: Supplementary Data 1

Description: We have uploaded the source data as Supplementary Data in Excel format.
