# Supplementary material for: Impact of gallbladder hypoplasia on hilar hepatic ducts in biliary atresia
Source: Commun Med (Lond). 2024 Jun 11;4:111. doi: 10.1038/s43856-024-00544-5 (PMC11166647; doi:10.1038/s43856-024-00544-5)
Supplement: Supplementary file 5 — Reporting Summary [file 43856_2024_544_MOESM5_ESM.pdf]

nature portfolio

Corresponding author(s): Yoshiakira Kanai

Last updated by author(s): Mar 31, 2024

Reporting Summary

Nature Portfolio wishes to improve the reproducibility of the work that we publish. This form provides structure for consistency and transparency in reporting. For further information on Nature Portfolio policies, see our Editorial Policies and the Editorial Policy Checklist.

Statistics

For all statistical analyses, confirm that the following items are present in the figure legend, table legend, main text, or Methods section.

n/a	Confirmed	
☐	☒	The exact sample size (*n*) for each experimental group/condition, given as a discrete number and unit of measurement
☐	☒	A statement on whether measurements were taken from distinct samples or whether the same sample was measured repeatedly
☐	☒	The statistical test(s) used AND whether they are one- or two-sided<br>*Only common tests should be described solely by name; describe more complex techniques in the Methods section.*
☐	☒	A description of all covariates tested
☐	☒	A description of any assumptions or corrections, such as tests of normality and adjustment for multiple comparisons
☐	☒	A full description of the statistical parameters including central tendency (e.g. means) or other basic estimates (e.g. regression coefficient) AND variation (e.g. standard deviation) or associated estimates of uncertainty (e.g. confidence intervals)
☐	☒	For null hypothesis testing, the test statistic (e.g. *F*, *t*, *r*) with confidence intervals, effect sizes, degrees of freedom and *P* value noted<br>*Give P values as exact values whenever suitable.*
☒	☐	For Bayesian analysis, information on the choice of priors and Markov chain Monte Carlo settings
☒	☐	For hierarchical and complex designs, identification of the appropriate level for tests and full reporting of outcomes
☐	☒	Estimates of effect sizes (e.g. Cohen's *d*, Pearson's *r*), indicating how they were calculated

Our web collection on statistics for biologists contains articles on many of the points above.

Software and code

Policy information about availability of computer code

Data collection	Image data were obtained by using by fluorescence microscopy (BX51N-34-FL-2 , SZX12, SZX16 or Leica TCS SP8 confocal laser microscopy. The 3D images were obtained by using ImageJ FIIJI software v. 1.53t/ Java 1.8.0_172 (National Institutes of Health, MD).Both PBD+ gut length , total gut length and PBD+ CBD diameter were measured using ImageJ FIIJI software v. 1.53t/ Java 1.8.0_172 (National Institutes of Health, MD) .The gallbladder-cystic duct length and the minimum diameter of the common bile duct and Liver degeneration area sere measured by ImageJ software v. 2.3.0 (National Institutes of Health, MD)
Data analysis	Statistical data were analyzed by Prism9 (GraphPad Software) and JMP Pro software v.16.0 (SAS Institute, NC).

For manuscripts utilizing custom algorithms or software that are central to the research but not yet described in published literature, software must be made available to editors and reviewers. We strongly encourage code deposition in a community repository (e.g. GitHub). See the Nature Portfolio guidelines for submitting code & software for further information.

Data

Policy information about availability of data

All manuscripts must include a data availability statement. This statement should provide the following information, where applicable:

- Accession codes, unique identifiers, or web links for publicly available datasets
- A description of any restrictions on data availability
- For clinical datasets or third party data, please ensure that the statement adheres to our policy

All data underlying the findings are available within the paper and its Supplementary data. Correspondence and requests for materials should be addressed to Yothiskira kanai (ykanai@g.ecc.u-tokyo.ac.jp).

Human research participants

Policy information about studies involving human research participants and Sex and Gender in Research.

Reporting on sex and gender	A total of 127 participants were enrolled for the experiments, with each participant contributing one sample. Out of the 127 participants, 68 were biologically female, while the remaining 59 were biologically male.
Population characteristics	Among the participants, 3 individuals were diagnosed with biliary atresia with splenic malformation. Among these, 2 participants were categorized into the "Others" group, while 1 participant was excluded due to the absence of gallbladder epithelia. An additional participant in the "Others" group was identified with severe congenital heart disease, and yet another participant in the same group had a cleft palate. The remaining 122 participants exhibited no abnormalities.
Recruitment	All patients who had undergone KASAI portoenterostomy for biliary atresia were included in the recruitment process. All potential participants either provided informed consent or were given the opportunity to decline participation through an opt-out approach. As a result, every eligible candidate chose to take part in this study.
Ethics oversight	The ethics committees of the participating institutes (Kyoto Prefectural University of Medicine, approval ID ERB-G-117, University of Tokyo, approval ID 2021060G; Saitama Prefectural Children's Medical Center, approval ID 2020-06-020; and National Center for Child Health and Development, approval ID 2021001) approved the study protocol.

Note that full information on the approval of the study protocol must also be provided in the manuscript.

Field-specific reporting

Please select the one below that is the best fit for your research. If you are not sure, read the appropriate sections before making your selection.

☒ Life sciences ☐ Behavioural & social sciences ☐ Ecological, evolutionary & environmental sciences

For a reference copy of the document with all sections, see nature.com/documents/nr-reporting-summary-flat.pdf

Life sciences study design

All studies must disclose on these points even when the disclosure is negative.

Sample size	Throughout this study, "n" refers to the number of embryos, animals and humans. Exact n values are provided in the Methods section and Figure legend in the manuscript. At least three distinct animals were used each analysis. We used as minimal animals as possible available for each experiment based our prior report. Because the experiments using human samples were exploratory research, we did not calculate the sample size beforehand.
Data exclusions	53 human gallbladder samples were excluded because of the missing of GB walls. Some mouse embryos were excluded from analysis due to injection or sampling error. These are: 1 embryos (wild:1, mutant: 0) in Fig 1b', 6 embryos(wild: 3, mutant: 3) in Fig 1c', 29 embryos in Fig2a' (wild: 19, mutant: 10) and 1 embryos (Sham:1, PBD:0) in Figure S1a'-"Liver weight".
Replication	The histological experiments were replicated 2-3 times in the animal model.
Randomization	For the analysis of mutant mice, randomization was not feasible as the genotypes of the mice needed to be determined. For the analysis of only wild -type mice, embryos were randomly allocated to both the injection and control groups. Since the experiments involving human samples followed a retrospective study design, randomization was not feasible.
Blinding	Scientists were blinded to group allocation during data collection and analysis of all experiments.

Reporting for specific materials, systems and methods

We require information from authors about some types of materials, experimental systems and methods used in many studies. Here, indicate whether each material, system or method listed is relevant to your study. If you are not sure if a list item applies to your research, read the appropriate section before selecting a response.

Materials & experimental systems

n/a	Involved in the study
☐	☒ Antibodies
☒	☐ Eukaryotic cell lines
☒	☐ Palaeontology and archaeology
☐	☒ Animals and other organisms
☐	☒ Clinical data
☒	☐ Dual use research of concern

Methods

n/a	Involved in the study
☒	☐ ChIP-seq
☒	☐ Flow cytometry
☒	☐ MRI-based neuroimaging

Antibodies

Antibodies used

- primary antibodies [format: host anti-protein (company, catalog number, dilution, lot number & Clone number if available)]
1. mouse monoclonal anti-aSMA/ACTA2 (Sigma, A5228, 1:100, clone 1A4)
2. Rhodamine dolichos biflorusagglutinin (Vector, RL-1032, 5-10µg/ml, ZF1031/ZH0114/ZI0124)
3. mouse monoclonal anti-CDH1 (BD Transduction lab, 610181, 1:250, 328535, clone 36/E-Cadherin)
4. rabbit polyclonal anti-GFP (MBL, 598, 1:200, 078/084)
5. mouse monoclonal anti-GFP(MBL, M048-3, 1:50, clone1E4)
6. goat polyclonal anti-SOX17 (R&D Systems, AF1924, 1:100)
7. rabbit polyclonal anti-SOX9 (Millipore, AB5535, 1:1000)

-secondary antibodies [format: host anti-protein (company, catalog number, dilution, lot number if available)]:
8. goat anti-mouse Alexa 488 (Abcam, ab150113, 1:100)
9. donkey anti-mouse Alexa 488 conjugated (Invitrogen, A-21202, 1:400,)
10. chicken anti-rabbit Alexa 488 conjugated (Invitrogen, A-21441, 1:400)
11. goat anti-mouse Alexa 594 conjugated (Invitrogen, A-11032, 1:400, 230112)
12. rabbit anti-goat IgG biotinylated (Vector Laboratories, BA-5000, 1:400)
13. goat anti-rabbit IgG biotinylated (Vector Laboratories, BA-1000, 1:400)

Validation

All the antibodies used in this study are commercially available, validated by the manufactures or by our laboratory:
1. Anti-aSMA/ACTA2 (https://www.sigmaaldrich.com/JN/en/product/sigma/a5228)
2. Rhodamine dolichos biflorusagglutinin (https://vectorlabs.com/products/rhodamine-dolichos-biflorus-agglutinin-dba)
3. Anti-CDH1 (https://www.bdbiosciences.com/en-ca/products/reagents/microscopy-imaging-reagents/immunofluorescence-reagents/purified-mouse-anti-e-cadherin.610181)
4. Anti-GFP (https://ruo.mbl.co.jp/bio/dtl/A/?pcd=598)
5. Anti-GFP (https://ruo.mbl.co.jp/bio/dtl/A/index.html?pcd=M048-3)
6. Anti-SOX17 (https://www.rndsystems.com/products/human-sox17-antibody_af1924)
7. Anti-SOX9 (https://www.merckmillipore.com/JP/ja/product/Anti-Sox9-Antibody,MM_NF-AB5535?ReferrerURL=https%3A%2F%2Fwww.google.com%2F)
8. Anti-mouse Alexa 488 conjugated (https://www.abcam.co.jp/products/secondary-antibodies/goat-mouse-igg-hl-alexa-fluor-488-ab150113.html
9. Anti-mouse Alexa 488 conjugated (https://www.thermofisher.com/antibody/product/Donkey-anti-Mouse-IgG-H-L-Highly-Cross-Adsorbed-Secondary-Antibody-Polyclonal/A-21202)
10. Anti-rabbit Alexa 488 conjugated (https://www.thermofisher.com/antibody/product/Chicken-anti-Rabbit-IgG-H-L-Cross-Adsorbed-Secondary-Antibody-Polyclonal/A-21441)
11. Anti-mouse Alexa 594 conjugated (https://www.thermofisher.com/antibody/product/Goat-anti-Mouse-IgG-H-L-Highly-Cross-Adsorbed-Secondary-Antibody-Polyclonal/A-11032)
12. Anti-goat IgG biotinylated (https://vectorlabs.com/products/antibodies/biotinylated-rabbit-anti-goat-igg)
13. Anti-rabbite igG biotinylated (https://vectorlabs.com/products/biotinylated-goat-anti-rabbit-igg)

Animals and other research organisms

Policy information about studies involving animals; ARRIVE guidelines recommended for reporting animal research, and Sex and Gender in Research

Laboratory animals

Housing condition: Animals were provided with water and commercial laboratory mouse chow ad libitum and were housed under controlled lighting conditions (daily light from 07:00 to 19:00) .
Species: Mus musculus
Strain: ICR, C57B6/BL, Sox17, Sox17; AlbCre;ROSA26tdTomato, ShhCre; Sox17flox/flox, Sox17-eGFP knock-in mice.
Age: E13.5-18.5

Wild animals

No wild animals were used in this study.

Reporting on sex

Male and female embryos were used for all in vivo experiments.

Field-collected samples

No field -collected samples were used in this study.

Ethics oversight

All animal experiments were performed in strict accordance with the Guidelines for Animal Use and Experimentation of the University of Tokyo. All procedures were approved by the Institutional Animal Care and Use Committee of the Graduate School of Agricultural and Life Sciences at the University of Tokyo (approval ID P13-763, P14-877, P18-121 and P20-035).

Note that full information on the approval of the study protocol must also be provided in the manuscript.

Clinical data

Policy information about clinical studies

All manuscripts should comply with the ICMJE guidelines for publication of clinical research and a completed CONSORT checklist must be included with all submissions.

Clinical trial registration

This study was not a clinical trial, and therefore, there was no registration involved.

Study protocol

The study protocol received approval from the ethics committees of the participating institutes: Kyoto Prefectural University of Medicine (approval ID ERB-G-117), University of Tokyo (approval ID 2021060G), Saitama Prefectural Children's Medical Center (approval ID 2020-06-020), and National Center for Child Health and Development (approval ID 2021001).

Data collection

A total of 115 gallbladder samples were collected during Kasai portoenterostomy. These samples comprised 8 cases from 2018 to 2021 at the University Hospital of Kyoto Prefectural University of Medicine, 70 cases from 2000 to 2021 at the Saitama Children's Medical Center, 5 cases from 2020 to 2021 at the National Center for Child Health and Development, and 32 cases from 1995 to 2021 at the University of Tokyo Hospital.

Outcomes

We did not predefine the outcomes.
